# Supplementary material for: Inflammatory factors and risk of lung adenocarcinoma: a Mendelian randomization study mediated by blood metabolites
Source: Front Endocrinol (Lausanne). 2024 Aug 27;15:1446863. doi: 10.3389/fendo.2024.1446863 (PMC11384989; doi:10.3389/fendo.2024.1446863)
Supplement: Supplementary file 1 [file DataSheet1.zip › supplementary files/S2/GCST90200956/sensitivity-analysis.pdf]

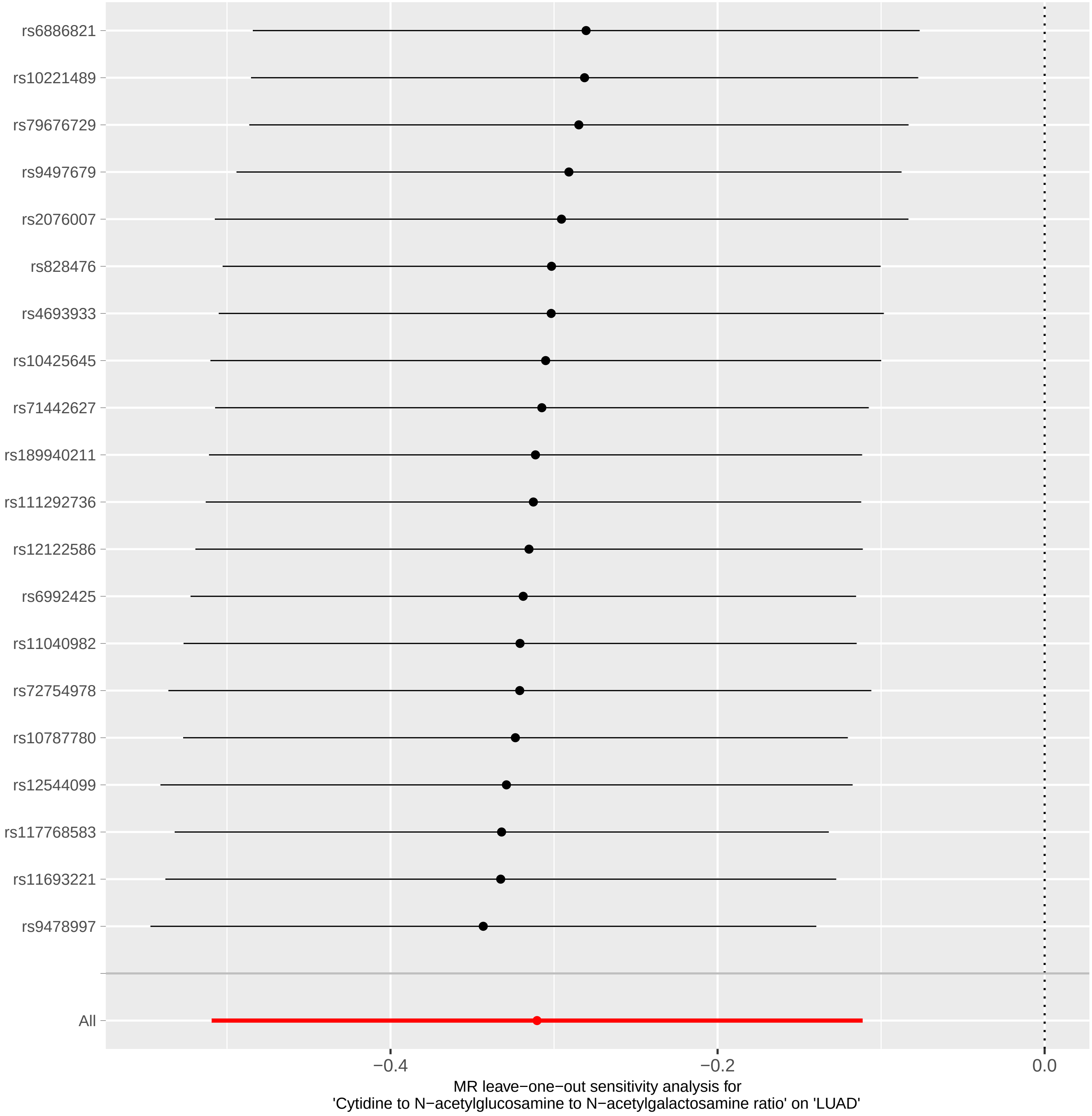

rs6886821
rs10221489
rs79676729
rs9497679
rs2076007
rs828476
rs4693933
rs10425645
rs71442627
rs189940211
rs111292736
rs12122586
rs6992425
rs11040982
rs72754978
rs10787780
rs12544099
rs117768583
rs11693221
rs9478997
All
−0.4
−0.2
0.0
MR leave−one−out sensitivity analysis for
'Cytidine to N−acetylglucosamine to N−acetylgalactosamine ratio' on 'LUAD'
